# Pathogenesis of Calcium Oxalate Kidney Stones, Interactions Among Mitochondria, NADPH Oxidase and Endoplasmic Reticulum

**DOI:** 10.3390/biom16101380

**Published:** 2026-09-22

**Authors:** Saeed R. Khan, Abdel A. Alli

**Affiliations:** 1 Department of Pathology, College of Medicine, University of Florida, Gainesville, FL 32610, USA; 2 Division of Nephrology, Hypertension and Transplantation, Department of Medicine, College of Medicine, University of Florida, Gainesville, FL 32610, USA; aalli@ufl.edu

**Keywords:** calcium oxalate, mitochondria, endoplasmic reticulum, ROS induced ROS release

## Abstract

Calcium oxalate (CaOx) nephrolithiasis is a common urological disease affecting people of all genders, age and races. Patients are generally treated surgically, prescribed medicine and advised to keep hydrated in addition to making dietary modifications. Despite advances, the stone recurrence rate has remained constant, between 30 to 50%. In addition, stones can lead to other renal diseases and even chronic kidney disease (CKD). Thus, a clearer understanding of the pathogenesis is paramount to devising improved treatment options. CaOx stones are the result of many factors including hyperoxaluria and CaOx crystals, both of which have been shown to cause the production of reactive oxygen species (ROS), development of oxidative stress (OS), and localized inflammation and fibrosis. Nicotinamide adenine dinucleotide phosphate (NADPH) oxidase, mitochondria and endoplasmic reticulum (ER) all play significant roles in ROS production. They have been extensively investigated, mostly on an individual basis, for their possible involvement in crystal-induced renal cellular injuries. ROS-induced ROS release (RIRR) is a concept of small bursts of ROS triggering further ROS formation in other organelles. We have reviewed the available data of ROS production by NADPH oxidase, ER and mitochondria and examined the implications of possible interactions among them. We propose that CaOx crystals activate the epithelial renin-angiotensin aldosterone system (RAAS) and NADPH oxidase, leading to the production of ROS, which causes mitochondrial dysfunction and ER stress. RIRR leads to sustained OS, stimulating inflammasomes and the release of proinflammatory interleukins, resulting in cell death and fibrosis. Osteogenic signaling pathways are initiated promoting the deposition of calcium phosphate (CaP), which can then result in the formation of Randall’s plaque, the nidus for idiopathic CaOx stones. Dead and dying cells promote the formation of crystal plugs, which can act as stone nidus at the papillary tips.

## 1. Introduction

Formation of stones in the kidneys, nephrolithiasis, is one of the major urological diseases. The incidents of stone formation appear to be increasing in all parts of the world, with parallel increases in costs of patient care [1,2]. The stone disease may also be associated with other kidney-related disorders, such as diabetes [3], hypertension [4], metabolic syndrome [5] and chronic kidney disease [6]. Great progress has been made in the surgical and medical management of nephrolithiasis [2]; however, recurrence rates remain high [7]. Traditional therapies involve stone removal followed by medications and dietary changes focusing upon decreasing mineral supersaturation in the urine to reduce chances of crystallization within the kidneys. Perhaps a better understanding of the initial steps, formation of plaques and/or plugs, is necessary to develop pathogenesis-based therapies and prevent recurrence. Clinical and experimental studies have provided evidence for the involvement of ROS and oxidative stress (OS) in the formation of kidney stones [8]. Reactive oxygen species (ROS) play an important part in normal cellular functions, regulating various biological functions. They are generally produced when and where needed. Increased production can, however, lead to oxidative stress, triggering pathological disorders [9]. Mitochondria and NADPH oxidases are major sources of ROS in the cells [10,11]. The endoplasmic reticulum is closely aligned with mitochondria, with high levels of hydrogen peroxide where it is produced as a byproduct of the protein folding process [12]. This article will review the possible participation of all three in the development of oxidative stress in renal tissues in response to exposure to oxalate (OX) and calcium oxalate (CaOx) crystals and involvement in the formation of the most common kidney stones, the CaOx stones.

## 2. Calcium Oxalate Kidney Stones

Kidney stones are solid crystalline masses with a small amount of organic matter mixed in. Most are made of CaOx mixed with varying amounts of calcium phosphate (CaP) [2]. Struvite, cystine, and uric acid are some of the other major stone types. Since urinary supersaturation leads to crystallization and stone formation, even some drugs and contaminants such as melamine, if present in urine in high quantities, can form kidney stones.

Urinary crystals and kidney stones include a small but pervasive matrix of lipids, proteins and carbohydrates. Results of one of the more recent studies using label-free proteomic methodology identified more than 1000 unique proteins in the organic matrix of CaOx kidney stones [13]. Proteins involved in inflammatory processes are common [14,15]. Origins of matrix proteins appear to be diverse, indicating kidney, blood and leukocyte sources [16]. Interestingly, a high degree of similarity of anionic proteins was found between organic matrices of human and cat stones [17]. Urinary macromolecules, which eventually become incorporated in growing stones as part of a matrix, take part in both crystallization as well as inflammation [8].

Kidney stones are generally found free or attached to the tubular or papillary surface epithelium [1,2]. They are formed through crystallization followed by crystal retention within the kidneys. The process is regulated by various promoters and inhibitors. Once formed, crystals must be retained within the kidneys. Reactive oxygen species impact both crystal formation and retention within the kidneys [8]. Crystallization can occur at a lower concentration in the presence of nucleators, and it has been shown that lipids of membrane fragments and vesicles are excellent nucleators of both CaOx and CaP crystals [18,19]. Animal models and cell culture studies have shown that exposure to both Ox and CaOx crystals results in epithelial injury and release of membrane fragments and vesicles [20]. Examination of human urine from kidney stone patients has also revealed the presence of phospholipids [21].

Results of animal model studies reveal that hyperoxaluria promotes upregulation of a variety of genes and production of proteins involved in crystallization and their retention [22,23,24]. There is an increase in the expression of crystallization modulators such as osteopontin (OPN), Tamm–Horsfall Protein (THP), bikunin, inter alpha inhibitor (IαI), α-1 microglobulin, prothrombin fragment-1 (PTF-1), calgranulin, heparin sulfate (HS), and Matrix Gla Protein (MGP). Oxidative stress may also be responsible for the posttranslational modifications of proteins, such as OPN controlling their crystallization behavior [25].

Crystal retention can be accomplished either by aggregation of crystals to form a mass big enough to block urinary flow through the tubule or by attachment to a platform, epithelia of the terminal collecting ducts or a plaque on the renal papillary surface [2,26,27]. Both methods of crystal retention were described by Randall [28]. Crystal retention in the terminal collecting ducts is now called Randall’s plug and on the papillary surface, Randall’s plaque. The plaque generally comprises apatite calcium phosphate. The plugs can also contain apatite [1]. Microscopic examination of renal crystal deposits in humans, rats and mice reveal them surrounded by membranous vesicles and cellular degradation products [29,30]. These components increase the size of the aggregates and thus help their retention within the tubules. Retention through attachment requires changes/injuries to the tubular epithelia, which appear to be caused by their exposure to high levels of oxalate and or CaOx crystals. Cell culture and animal model studies have shown that crystals adhere to injured epithelial cells. In addition, crystals can also attach to the basement membrane exposed by the loss of injured cells. The formation of both the plugs and plaques appears to involve renal injury and inflammation, with involvement of reactive oxygen species [8,31,32]. Calcium oxalate stones of patients with primary hyperoxaluria, those formed after bariatric surgery, and some idiopathic stones are found attached to Randall’s plugs [33]. Crystals of the plugs were surrounded by localized inflammation, fibrosis, injury and cell loss [29]. Areas surrounding Randall’s plaques also show necrotic tubules with thickened and layered basal lamina, in association with the CaP spherules embedded in a matrix of collagen and other fibers. In addition, the presence of such molecules as OPN, heavy chains of inter-α- inhibitor [34], and collagen in the interstitial CaP also suggest a role for inflammation in plaque formation.

Other signs of injury and inflammation in association with stone formation include urinary excretion of higher than normal levels of renal enzymes, gamma-glutamyl transpeptidase (GGTP), angiotensin-1-converting enzyme (ACE), beta-galactosidase (GAL), and N-acetyl-beta-glucosaminidase (NAG) in the urine of idiopathic CaOx stone formers [35]. Significantly increased NAG, β-galactosidase, glutathione S-transferase (GST), malondialdehyde (MDA) and thiobarbituric acid-reactive substances (TBARS) in the urine [36] also suggest that ROS were most likely responsible for the injury. An increase in urinary excretion of 8-hydroxydeoxyguanosine (8-OHdG), a marker of oxidative damage to DNA, was positively correlated with tubular damage as assessed by urinary excretion of NAG. Antioxidant deficiency was reported in idiopathic calcium stone formers with and without stones. Calgranulin, α-defensin, myeloperoxidase, and anti-inflammatory proteins were increased in the urine of stone patients as well as the inner core of the CaOx stones. Similarly, the IαI family of proteins, which are important participants in wound healing, were significantly elevated in male stone formers’ urine. They were also present in the stone matrix and the matrices of the CaOx and CaP crystals generated in the urine [36,37]. Hyaluronan was also a major constituent of the organic matrix of stones. Prothrombin fragment-1 was also identified in the urine and stone matrix and preferentially bound to CaOx crystals. The mRNA for MCP-1 as well as IL-6 were also identified in kidneys of stone formers.

## 3. Reactive Oxygen Species

Free radicals, atoms or molecules with unpaired electrons in their outer orbit, and their metabolites, are highly reactive and are called Reactive Oxygen Species. They play a critical role as signaling molecules and mediators in a variety of regulatory processes and pathways including proliferation, activation or inactivation of regulatory biomolecules, and regulation of transcriptional activities. The major cellular ROS include superoxide anions (O_2_^−^•), nitrogen dioxide radicals (NO_2_•), and hydroxyl radicals (OH•). Other ROS with oxidizing properties, but are not free radicals, include hydrogen peroxide (H_2_O_2_), peroxynitrite (ONOO^−^), and hypochlorous acid (HOCL). They are produced through a variety of processes (Figure 1). Superoxide anions (O_2_^−^•) are byproducts of the mitochondrial respiratory chain as well as activities of NADPH oxidase, xanthine oxidase, lipoxygenase, cyclooxygenase, and hem oxygenase. They may also be produced by lipid radicals. The eNOS can also produce O_2_^−^•. NO• radicals are produced through endothelial nitric oxide synthase (eNOS)-mediated oxidation of L-arginine. The interaction between superoxide and nitric oxide produces highly reactive peroxynitrite, ONOO^−^. Normally, generation of O_2_^−^•, NO• and their metabolites is strictly controlled and occurs at a steady state level, produced when needed and cleared through the actions of various scavengers and antioxidants. They are short-lived and do not travel away from where they are needed. ROS such as H_2_O_2_ activate signaling pathways like mitogen-activated protein kinase (MAPK) and phosphoinositide 3-kinase (PI3K)/protein kinase B (Akt). ROS activate transcription factors like nuclear factor erythroid 2-related factor 2 (Nrf2), hypoxia-inducible factor 1α (HIF-1α), activator protein 1 (AP-1), and nuclear factor kappa-light-chain-enhancer of activated B cells [38,39].

Excessive production of ROS leads to extensive damage to proteins, lipids and DNA [40]. Exposure of proteins to ROS leads to their misfolding. Membranes are damaged when their lipids undergo peroxidation. Lipid peroxide radicals trigger further oxidative damage to the lipids. Free radicals, in particular hydroxyl radicals, attack both nuclear and mitochondrial DNA. Mitochondrial injury causes opening of the permeability transition pores, loss of membrane potential, halting ATP production and release of cytochrome c. To combat ROS, the oxidants, and their overproduction, cells are equipped with several scavenging systems, the antioxidants (Figure 1). The imbalance between oxidants and antioxidants leads to oxidative stress (OS). Most cells respond to OS by boosting the levels of intracellular antioxidants. Antioxidants include superoxide dismutase (SOD), which eliminates O_2_^−^•, releasing less toxic H_2_O_2_. Glutathione (GSH) peroxidase (GPx) and catalase then detoxify H_2_O_2_, producing water. In a more complex transition metal-catalyzed reaction called the metal-catalyzed Haber–Weiss reaction, H_2_O_2_ yields an even more reactive hydroxyl radical, which is, however, short lived and works at short range. Initially, superoxide anions donate single electrons to ferric ions, resulting in molecular oxygen and ferrous ions. The Fenton reaction between ferrous ions and H_2_O_2_, leads to the formation of OH•. Mitochondria and NADPH oxidase are major endogenous sources of ROS (Figure 2) [11,41].

The role of ROS-induced injury and inflammation in stone formation has been investigated using tissue culture and animal models. The response of renal epithelial cell exposure to Ox and/or CaOx or CaP crystals is cell specific and time and concentration dependent. Both oxalate (Ox) and CaOx crystals are injurious to renal epithelial cells. Toxicity is linked to the production of free radicals [42] and lipid peroxidation [43,44].

Longer duration and higher concentrations are injurious, and cells of proximal tubular origin are more susceptible than cells of more distal sections of the nephron. The crystals adhere to the cells and are then internalized by the cells. Exposure to COM crystals leads to upregulation of genes encoding transcriptional factors, growth factors and extracellular matrix. Free radicals play a significant role in responding to the Ox and crystal exposure. Epithelial cells secrete superoxide when exposed to CaOx crystals. Injury is ameliorated by antioxidants and free radical scavengers [44]. Exposure to Ox, even at physiological concentrations, increases injury to the antioxidant-depleted LLC-Pk1 cells as determined by increased leakage of lactate dehydrogenase and cellular H_2_O_2_ [44]. Treatment with α-tocopherol and ascorbic acid restores the antioxidant levels of the exposed cells. Free radical scavengers, catalase and superoxide dismutase, also provide protection from oxalate-induced injury to normal LLC-PK1 and MDCK cells [45]. Catalase treatment of NRK52E renal epithelial cells exposed to Ox, CaOx, CaP or uric acid reduces MCP-1 mRNA and protein.

Several animal models, mostly rats and mice, have been developed to understand the pathogenesis of CaOx nephrolithiasis [46,47]. All of them require a production of hyperoxaluria by administration of Ox or its precursors. They lead to deposition of CaOx crystals in the kidneys. There is production of ROS, development of oxidative stress (OS) and renal epithelial injury, as detected by immunohistochemical examination of kidneys and analysis of the urine. Crystals form in tubular lumens and then may move into the interstitium following the loss of tubular epithelium. The mitochondria, endoplasmic reticulum and NADPH oxidase play significant roles [48,49,50,51]. Treatment of hyperoxaluric animals with antioxidants and inhibition of inflammatory pathways led to a reduction in crystal deposition, cellular injury, expression of upregulated genes, as well as production of macromolecules involved in crystallization and inflammation [52,53]. There was an increase in lipid peroxides in both the renal tissue and urine of hyperoxaluric rats. Treatment with vitamin E improved the levels of antioxidant enzymes in the kidneys, reduced injury and eliminated CaOx crystal deposition. Injury and inflammation were caused through the activation of NLR family pyrin domain containing 3 (NLRP3) inflammasome [54,55]. Results of another study showed increased expressions of genes for SOD, GPx, GST, aldehyde dehydrogenase, and mitochondrial uncoupling protein, and ceruloplasmin was increased in hyperoxaluric rats. Apocynin, an antioxidant and a non-specific inhibitor of NADPH oxidase completely reversed the effect of hydroxyproline-induced hyperoxaluria in [56]. Renal deposition of CaOx crystals was also appreciably reduced. A large number of animal studies have been performed to determine therapeutic use of herbal medicines. The commonly used herbal medicines have antioxidant and anti-inflammatory properties [57,58,59].

Citrate is also involved in maintaining endogenous antioxidant defenses. Administration of exogenous citrate to LLC-PK1 and MDCK cells bolstered these defenses and diminished the cellular injury inflicted by exposure to increased Ox and CaOx crystals [60]. The presence of citrate in the culture medium was associated with a significant increase in glutathione (GSH) peroxidase and a drop in the production of H_2_O_2_ and 8-isoprostane (8-IP), which is an end product of lipid breakdown. There was a significant improvement in cell viability as demonstrated by decreased LDH release and increased trypan blue exclusion.

## 4. Mitochondrial Involvement in the Production of Reactive Oxygen Species

Mitochondria are both a producer and a target for ROS. They are generally the most common source of superoxide and H_2_O_2_. The formation of superoxide (O_2_^−^) in both the mitochondrial matrix and the intermembrane space occurs at mitochondrial electron transport chain (ETC) complexes I and III [61,62] during ATP production. Superoxide dismutase 1 and 2 catalyze the conversion of O_2_^−^ into H_2_O_2_ in the intermembrane space and matrix, respectively. Incomplete reduction of oxygen leads to the formation of O_2_^−^. It can also be produced through reverse electron transport.

One of the earlier studies determined the source of superoxide, by exposing renal epithelial cells in culture to COM crystals [63]. Crystal exposure increased superoxide production and decreased glutathione. Using selective inhibitors and a variety of mitochondrial probes, they confirmed that mitochondria were responsible for superoxide production. Exposure of renal epithelial cells in culture to Ox alone decreased mitochondrial membrane potential attenuated by an inhibitor of cytosolic phospholipase A2 (PLA2). Isolated renal mitochondria also responded to oxalate, arachidonic acid, lyso-PC and ceramide by accumulating ROS, lipid peroxides and thiol proteins [64]. Another study investigated changes in the mitochondrial proteome of MDCK cells induced by COM crystal internalization [65]. Fifteen proteins involved in cellular structure, carbohydrate metabolism and energy metabolism were significantly altered. Oxidatively modified mitochondrial proteins were markedly increased.

Mitochondrial damage is suggested to be induced by the opening of the mitochondrial permeability transition pore (mPTP). mPTP opening depends upon the activation of cyclophilin D in the mitochondrial matrix by ROS produced by NADPH oxidase and is inhibited by cyclosporine A (CSA) [66]. CSA prevented the depolarization of mitochondrial membrane, decreased SOD expression, increased 4-hydroxy-2-nonenal (4HNE) and released cytochrome-c into the cytosol in NR52E renal epithelial cells exposed to CaOx monohydrate crystals in vitro. CSA treatment of hyperoxaluric rats resulted in reduced mitochondrial damage, oxidative stress and CaOx crystal deposition in the kidneys. Another study of mitochondrial dysfunction in rats with hyperoxaluria and CaOx crystal deposition was seen as decreased activities of electron transport chain complexes I, II, IV; increased mitochondrial ROS, openings of the mPTP and mitochondrial swelling [67]. The trend could be reversed by apocynin and N-acetyl cysteine treatment. Another cell culture study investigated a mitochondria-specific antioxidant, mitoTEMPO, for its impact on oxalate-induced injury. Pre-treatment of NRK52E, a proximal tubular cell line, decreased mitochondrial ROS production, release of LDH and malondialdehyde (MDA) and expression of IL-6 and OPN. In addition, mitochondrial membrane potential was restored and expression of Nox4 and p22 proteins were altered [48,68]. Treatment with taurine, which has been shown to prevent oxidative injury of the mitochondria, reversed mitochondrial changes in hyperoxaluric rat kidneys and decreased crystal deposition [69].

Exposure of isolated rat kidney mitochondria to COM crystals produced a dose-dependent increase in mitochondrial swelling and decrease in State 3 respiration [70]. Rat kidneys in a model of CaOx crystalluria were under oxidative stress associated with a decrease in mitochondrial GSH concentration and increased activity of glucose-6-phosphate dehydrogenase (G6PDH). There was a decrease in mitochondrial cytochrome c and oxygen consumption [71]. Exposure of MDCK cells to COM crystals and their internalization led to changes in the mitochondrial proteome [65]. Significantly altered proteins were related to various biological functions. There was an increase in oxidatively modified mitochondrial proteins, demonstrating a loss of ROS regulation by mitochondria.

It has been hypothesized that kidneys are capable of actively removing interstitial crystal deposits through macrophage activities [72,73,74]. Our in vitro studies have demonstrated that activated macrophages are able to destroy both stone fragments and synthesized CaOx crystals through clathrin-mediated endocytosis and phagocytosis [74]. We have also shown that both epithelial cells and macrophages produce monocyte chemoattractant-1 (CCL2) in response to an exposure to CaOx crystals [75,76,77]. Treatment with antioxidants reversed these responses. Genes encoding proinflammatory macrophages are upregulated whereas those of anti-inflammatory macrophages are downregulated in the kidneys of stone patients [78,79,80]. Monocytes derived from stone formers showed decreased mitochondrial function [81]. Exposure of THP-1 cells, a human monocyte-derived cell line, to varying concentrations of CaOx crystals and sodium oxalate induced mitochondrial dysfunction and redox imbalance [82]. Dietary oxalate also impacts monocyte metabolism and immunological response [83]. RNA sequencing of isolated monocytes after oxalate overload showed modifications in several pathways, including mitochondrial metabolism. Exposure of monocytes and macrophages to oxalate in vitro triggered oxidative stress, mitochondrial and lysosomal dysfunction, decline in IL-10, and impaired autophagy. Administration of exogenous IL-10 and MitoQ mitigated all these reactions [84]. Results of such studies demonstrate involvement of mitochondria in monocyte/macrophage activities in response to oxalate.

Hyperoxaluria impacts mitochondria through cellular exposure to oxalate and/or CaOx crystals. Oxalate enters the cells through various transporters and can directly meet cellular organelles. It effects mitochondrial membrane potential and ATP production, with both going down. The electron transport chain is damaged, causing electron leakage. Leaked electrons react with oxygen to convert into superoxide. Superoxide is converted into hydrogen peroxide through the activity of the mitochondrial superoxide dismutase. Fenton reaction leads to the formation of hydroxyl radicals. Nitric oxide action leads to the formation of per-oxynitrate. Increased production of mitochondrial ROS damages mitochondrial DNA, proteins, and lipids, leading to mitochondrial dysfunction. Cytochrome c is released into the cytoplasm, activating a chain of caspases resulting in apoptosis [85,86]. Pyroptosis, another form of death, also involves mitochondrial dysfunction [87]. Mitochondrial ROS produced in response to high oxalate act as a danger signal. Hyperoxaluria in rats produced upregulation of genes encoding for PYCARD (ASC: apoptosis-associated spec-like protein containing a CARD), TXNIP (Thioredoxin-interacting protein), NLRP3 inflammasome, Caspase-1, IL-1β and IL-18 [55]. ROS involvement in NLRP3 formation and pyroptotic death of HK2 cells exposed to oxalate, was inhibited by GSDMD inhibitor NSA. Hyperoxaluria in GSDMD−/− mice produced appreciably a smaller number of crystals than wild-type mice [88]. Both COM and COD crystals upregulated the expressions of NLRP3, caspase-1, GSDMD-N and IL1B [89]. Mitochondria also appear to be involved in ferroptosis. Cells undergoing ferroptosis had smaller mitochondria with reduced number of cristae [87].

## 5. NADPH Oxidase-Induced Production of Reactive Oxygen Species

Nicotinamide adenine dinucleotide phosphate (NADPH) oxidase (NOX) is a family of membrane enzymes involved in the generation of ROS. They are in the plasma membrane as well as mitochondria, endoplasmic reticulum and focal adhesions. There are seven isoforms, of which NOX1, NOX2 and NOX4 are expressed in the kidneys and differ in their location and regulatory requirements. NOX2 complex consists of the membrane subunits gp91phox and p22phox, along with the regulatory cytosolic subunits p67phox, p47phox, p40phox, and the small GTPases protein, RAC [90]. Activation requires stimulation of protein kinase c (PKC), which phosphorylates cytosolic p47-phox. Cytosolic units and G-protein Rac translocate to the membrane. NOX4 is the predominant NADPH oxidase in the kidneys. It is enriched in the ER, mitochondria-associated membrane, and nuclear membrane. While NOX2 produces superoxide, NOX4 produces H_2_O_2_.

Epithelial exposure to Ox/CaOx crystals most likely starts with the activation of NADPH oxidase [23,91,92]. We have investigated hyperoxaluria-induced NADPH oxidase activation and production of ROS. Detailed results have been presented in our earlier publications [93,94]. NADPH oxidase is a major source of ROS in the presence of angiotensin II [95,96]. All components of the renin-angiotensin system (RAS) are produced by the kidneys and play a major role in renal diseases [97]. Renin acts to cleave liver-made angiotensinogen to make angiotensin I (Ang I). Angiotensin-converting enzyme (ACE) converts Ang I to Ang II. Ang II acts though two receptors, AT1 and AT2. Ang II is implicated in causing oxidative stress by stimulating membrane-bound NADPH oxidase, leading to increased generation of superoxide [98]. A significant reduction in the production of renal lipid peroxides and expression of TGF-beta1 was seen in hyperoxaluric rats after administration of AT1 receptor blockers (ARB) or ACE inhibitors [99,100]. In addition, the administration of ARB to hyperoxaluric rats was associated with reduction in CaOx crystal deposition and expression of OPN and MCP1, pointing to an involvement of the renin-angiotensin system (RAS) in CaOx nephrolithiasis. TGF-beta participates in ROS production through NADPH oxidase activation [101,102]. Increased production of TGF-beta1 in Ox-induced generation of ROS in LLC-PK1 cells was significantly reduced by treatment with neutralizing TGF-beta antibodies [103]. In addition to activation of NADPH oxidase, results also showed that oxalate significantly increased membrane translocation of Rac1, which was stopped by selective inhibition of Rac1 [104]. Aldosterone is also involved in the oxalate/CaOx-induced activation of Nox through mineralocorticoid receptor (MR), Rac 1 and MAPK. Administration of spironolactone, a competitive antagonist of MR, to hyperoxaluric rats reversed the effect of hyperoxaluria on upregulated genes including those encoding for AGT, angiotensin receptors 1b and 2, Renin1, Nox 4 and Rac 1 [105]. Incubation of NRK-52EA cells in culture with CaOx crystals or aldosterone showed that both crystals and aldosterone activated Nox and Rac1. Spironolactone inhibited Nox and Rac1 expression. Increased Rac-1 expression was significantly tempered with exposure to PP2, a selective inhibitor of the SRC family of kinases. Many investigations have employed DPI and apocynin, which are shown to possess limited Nox specificity. Oxalate exposure was also associated with a significant increase in membrane-associated protein-kinase C (PKC) activity, while lowering cytoplasmic PKC activity [92]. Pretreatment of cells with specific inhibitors of PKC-alpha and delta blocked oxalate-induced NADPH oxidase activity, LDH release, lipid hydroperoxide and apoptosis.

## 6. Endoplasmic Reticulum Involvement in Oxalate-Induced Injury and Inflammation

The endoplasmic reticulum (ER) plays a significant role in cellular functions including protein synthesis, folding and transport, biosynthesis of lipids, calcium storage and redox homeostasis. Accumulation of unfolded proteins leads to ER stress through the unfolded protein response (UPR), impacting on normal functions, activating a variety of inflammatory pathways causing many metabolic diseases [106,107]. ER under stress also accumulate ROS-creating oxidative stress [108].

The results of several studies have provided evidence for the involvement of endoplasmic reticulum (ER) in hyperoxaluria-induced renal injury and inflammation. Transcriptome sequencing investigations of HK-2 cells exposed to CaOx crystals and human renal tissues from human kidney stone formers found a significant increase in the expression of genes related to ER stress and the unfolded protein response [50]. Hyperoxaluric rats with all marks of mitochondrial and NADPH oxidase involvement in the production of ROS also showed signs of ER stress [109]. Levels of glucose regulated protein 78 (Grp78), a marker of ER stress, were significantly increased in hyperoxaluric rats. Treatment with 4-phenylbutyric acid (4-PBA), an inhibitor of ER stress led to significant reduction in ROS levels. HK-2 cells exposed to CaOx crystals showed increasing expression of ER stress marker proteins GRP 78 and CHOP with increasing concentration of crystals. Pretreatment with 4-PBA reduced marker proteins as well as IL-1B and LDH release [110]. Another study investigated ER stress in oxalate-induced ferroptosis [111]. Both cell culture and mice models were employed. Glutathione-specific gamma-glutamylcyclotransferase 1 (CHAC1), a key enzyme catalyzing the degradation of glutathione (GS) and a specific symbol for ferroptosis, was used as one of the markers. Hyperoxaluria was induced by daily injection of glyoxylic acid for 14 days. 4-PBA was used as an inhibitor of ER stress. Several analytical techniques were employed. Results showed that 4-PBA treatment and CHAC1 deficiency both attenuated oxidative damage, decreased crystal deposition and improved kidney function.

## 7. Discussion

Using mostly PubMed and Google, we reviewed literature specific to ROS involvement in various human diseases as well as those that are specifically produced in response to hyperoxaluria and kidney stones. We selected those articles that are directly related to the concept of possible interactions between various sources of ROS. The majority of the studies involve cell culture and animal models. There is no animal model that replicates all the subtleties of human stone pathogenesis. Most animal models produce crystal depositions in the kidneys. Cell lines used differ in their origin, with respect to both nephron segments and animal species. Crystals the cells are exposed to are different from the crystals formed in the urinary environment. The chemicals used to understand the pathways involved have been shown to be active in experimental situations and not proven to be in humans.

It is apparent that mitochondria, endoplasmic reticulum and NADPH oxidase, all are involved in the production of ROS when renal epithelial cells in culture or in vivo become exposed to oxalate/CaOx crystals. Do they work in tandem or in production of ROS by one activating the other? Several investigations have examined interactions among the various ROS-producing cellular units. Interestingly ROS produced by the mitochondria may cause opening of the mitochondrial permeability transition pore (mPTP), releasing them into the cytosol, a process called ROS-induced ROS release (RIRR) [112]. RIRR may start a chain reaction leading to mitochondrial injury and eventually cell death. Evidence for RIRR in various model systems is provided in Table 1.

The results of several studies indicate that NADPH oxidase-induced ROS stimulate mitochondrial ROS production [120]. ROS produced by activation of NADPH oxidase promote mitochondrial ROS production through the renin-angiotensin-aldosterone system [121]. Ang II-mediated mitochondrial dysfunction was prevented by Nox inhibitor apocynin and PKC inhibitor chelerythrine. Depletion of p22phox, an essential component of NADPH oxidase, caused considerable reduction in ROS production in mitochondria isolated from Ang II-treated cells [122]. Mitochondrial ROS have also been shown to affect ROS production by NADPH oxidase. Exposure of Ang II-treated aortic endothelial cells to mitochondrial-targeted mitoTEMPO decreased mitochondrial superoxide and reduced cellular NADPH oxidase [123]. Depletion of Nox 2 in gp91phox knockout mice inhibited Ang II-induced cellular and mitochondrial superoxide [124]. As we presented earlier, the renin-angiotensin system is also involved in hyperoxaluria-induced oxidative stress, which can be prevented by treatment with NADPH oxidase inhibitors or Ang II receptor blockage.

Mitochondria and ER are physically connected through mitochondria-associated membranes (MAMs), and crosstalk between them plays a significant role in many diseases [125]. NOX4 is involved in regulating calcium transfer at MAMs [126], which in turn drives mitochondrial ROS production (Figure 3). Thus, damage to mitochondria can directly impact ER. ROS produced by mitochondria or NADPH oxidase or other sources impact several ER functions, the major being increases in protein misfolding and release of calcium. Overproduction of misfolded proteins leads to ER stress. Mitochondrial calcium overload markedly increases mitochondrial ROS. ER under stress generates more ROS, eventually producing oxidative stress, cell injury and death [127]. An interaction between ER stress and mitochondrial dysfunction during hyperoxaluria-induced oxidative stress was studied in a rat model [128]. Kidneys of the hyperoxaluric rats showed all signs of developing oxidative stress and CaOx nephrolithiasis. Hyperoxaluric rats were also treated with an inhibitor of ER stress, 4 phenylbutyric acid (4PBA), as well as a well-recognized antioxidant, N-acetyl cysteine (NAC). Inhibition of ER stress by 4PBA prevented a decrease in mitochondrial membrane potential and increase in mitochondrial calcium. NAC had a nominal effect on ER stress and mitochondrial dysfunction.

Small bursts of ROS from one source, triggering production of additional ROS from same or other source, causing a self-amplifying cycle resulting in oxidative damage, is called ROS-induced ROS release (RIRR). The term was initially applied to mitochondria, where initial production of ROS by mitochondria initiates opening of the mitochondrial permeability transition pore (mPTP) and inner membrane anion channels. This leads to increased generation of ROS by the electron transfer chain and their release into the cytosol, triggering RIRR [112]. The chain reaction leads to oxidative stress and eventual cell death. This appears to happen during hyperoxaluria. Hyperoxaluria-induced oxidative stress in the kidneys is the result of crosstalk among NADPH oxidase, mitochondria, and endoplasmic reticulum. A quick burst of ROS, because of an exposure to Ox or CaOx crystals, starts a chain reaction generating excess ROS, leading to OS.

ROS perform an important part in the activation of nucleotide-binding oligomerization domain (NOD)-like receptor protein-3 (NLRP3) inflammasome, triggering a release of proinflammatory cytokines IL-1B and IL-18, producing a robust inflammatory response [129,130]. We demonstrated an increase in the expressions of NLRP3, ASC, TXNIP, Caspase-1, IL-1β, and IL-18 in hydroxyproline-fed hyperoxaluric rats. The expression was reduced in the apocynin (an inhibitor of NADPH oxidase)-treated group [55]. Thioredoxin-interacting protein (TXNIP), interacts with thioredoxin (TRX), a major antioxidant protein, and inhibits its antioxidant function and expression [131]. The NLRP3 inflammasome plays a significant role in pathological vascular calcification [132], which requires smooth muscle cells acquiring an osteogenic phenotype [133,134,135]. Osteogenic signaling pathways involving bone morphogenetic proteins BMP 2 and BMP 4, and Wnt, are activated through upregulation of the transcription factors Runt-related transcription factor 2 (RUNX2), Osterix and msh homeobox 2 (MSX-2) [136,137]. Our animal model study has also shown osteogenic changes in the kidneys of hyperoxaluria rats [22]. Genes for RUNX1 and 2, zinc finger protein Osterix, bone morphogenetic proteins (BMP2 and 7), bone morphogenetic protein receptor (BMPR2), collagen, osteocalcin, osteonectin, OPN, MGP, osteoprotegerin (OPG), cadherins, FN and vimentin (VIM) were upregulated, while those for cytokeratins 10 and 18 were downregulated. Cytokeratin 8, 10, 18, and E-cadherin are expressed by epithelial cells, while N-cadherin, fibronectin and vimentin are expressed by mesenchymal cells.

Oxalate can directly enter the epithelial cells by diffusion or through anion transporters and interact with mitochondria-producing ROS. CaOx crystals forming in the tubular fluid, on the other hand, activate the epithelial renin-angiotensin-aldosterone system and NADPH oxidase, leading to the production of ROS [97,99,105] that can act on mitochondria, causing their dysfunction and on ER, causing ER stress [50,109]. Mitochondrial dysfunction includes decreased mitochondrial membrane potential and glutathione and increased cytochrome c and mitochondrial ROS. In addition, mitochondria contain specialized calcium transport systems that allow for electrogenic calcium uptake that in turn communicates with other pathways including ROS [138]. There is protein misfolding and calcium release from ER. Release of chemokines and cytokines following the activation of MAPK, NF-kB, and AP-1 transcription attract macrophages and leukocytes [139,140,141,142]. ROS-induced ROS release leads to sustained oxidative stress and activation of the inflammasome [142,143], which is followed by the release of proinflammatory interleukins. Osteogenic signaling pathways are initiated. There is fibrosis and cell death through apoptosis, pyroptosis or ferroptosis. All of these modes of cell death produce membrane-bound vesicles capable of promoting nucleation of crystals and localized calcification. CaP crystallization within the renal interstitium and outward growth of the calcification leads to the development of Randall’s plaque. Crystal formation in the tubular lumen and their retention produces crystal plugs. Under proper supersaturation, both the plaques and plugs become nidi for future stones.

## 8. Concluding Remarks

There is sufficient experimental and clinical data available to conclude that Ox and CaOx crystals can induce oxidative stress within the kidneys, with localized immunological and inflammatory responses. NADPH oxidase, mitochondria and the endoplasmic reticulum all play significant roles in ROS production. It is now well documented in other systems that ROS production at one site can trigger additional ROS production elsewhere through a self-amplifying, feedforward mechanism, the so-called RIRR. We are proposing that similar phenomena may be active in hyperoxaluric kidneys through the interactions among NADPH oxidase, mitochondria and endoplasmic reticulum. Small, localized changes in the urinary environment can start a self-sustaining cycle of ROS production, with obvious consequences of localized immunological and inflammatory responses. Several studies have been carried out to investigate the individual organelles involved in ROS production in response to hyperoxaluria. It is time to study the interactions among them and determine the starting point of the cycle so that treatment for stone recurrence can be found.

## Figures and Tables

**Figure 1:**
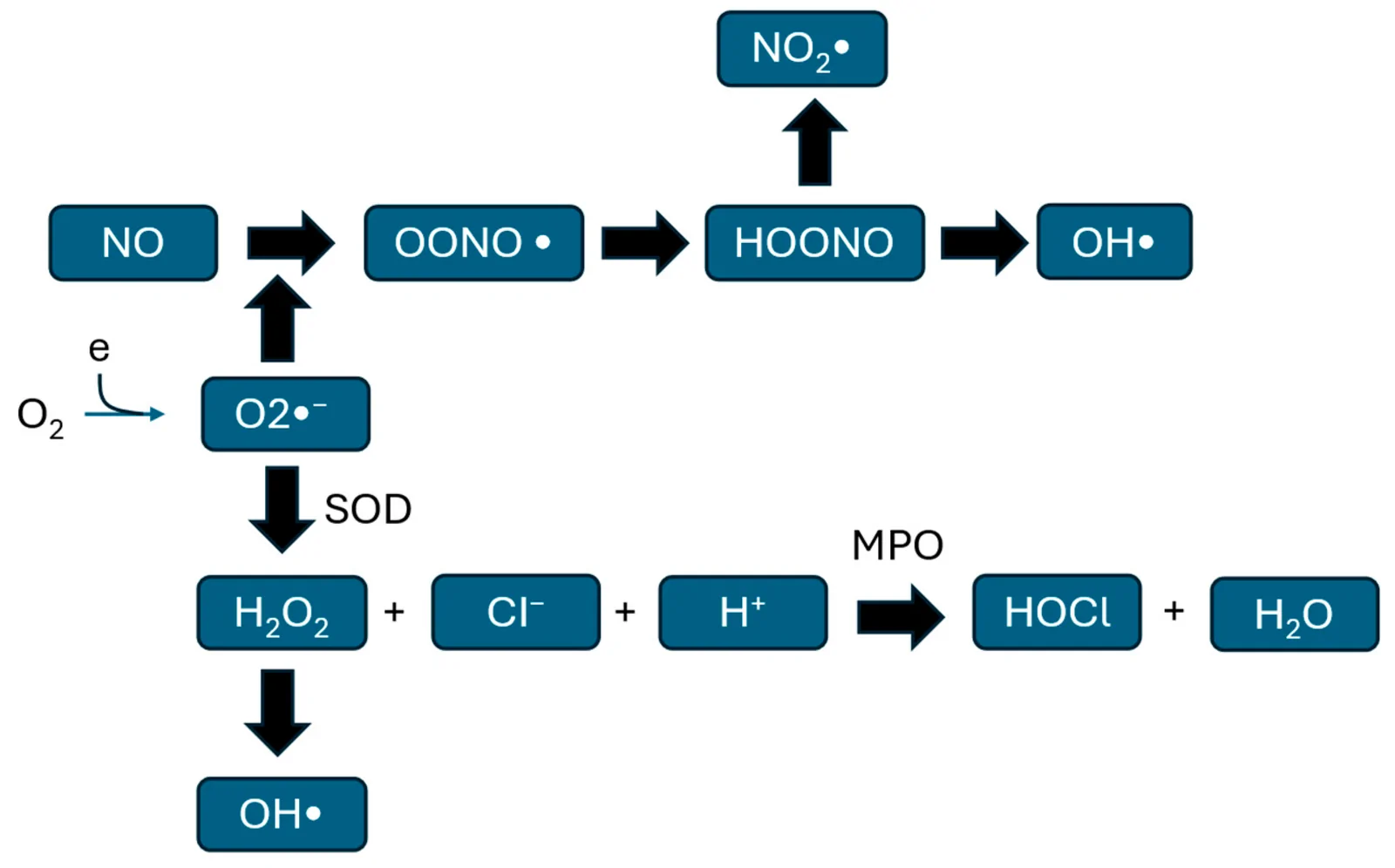
Reactive oxygen and nitrogen species. Multiple reactive oxygen species are formed from the transfer of one electron to oxygen through the superoxide anion radical O_2_^−^•. Superoxide dismutase (SOD) catalyzes the dismutation of O_2_^−^• to H_2_O_2_. OH• reacts instantaneously with molecules that attract hydrogen atoms. Myeloperoxidase (MPO) catalyzes the conversion of H_2_O_2_ and Cl^−^ to hypochlorous acid (HOCL), a strong oxidant that can function pathologically as an inflamogen. Also, nitrogen dioxide radicals (NO_2_•) originate from nitric oxide (NO).

**Figure 2:**
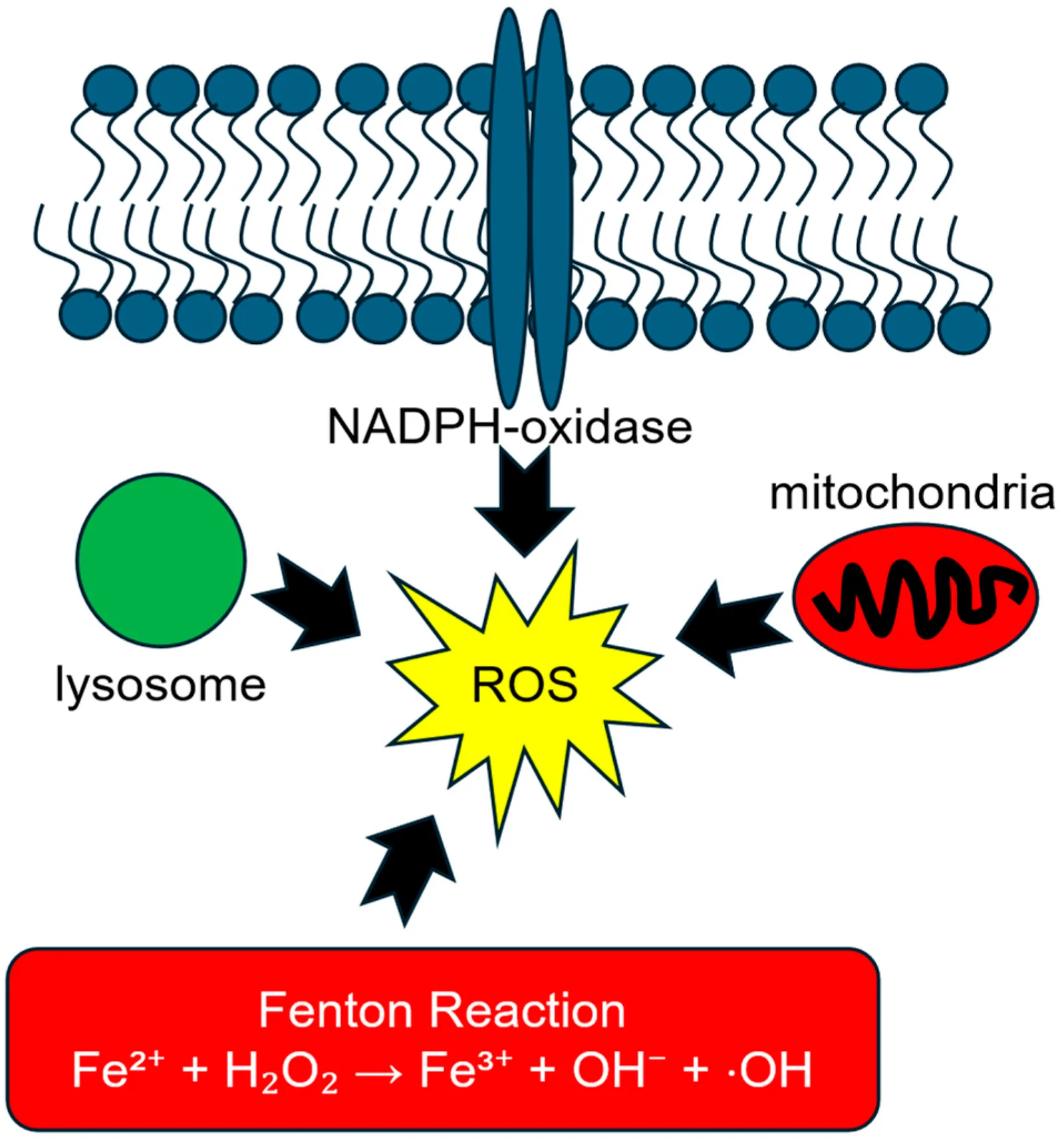
Different sources of intracellular ROS. Transition metals such as iron can mediate ROS production through the Fenton reaction. Lysosome membrane permeabilization (LMP) can result in ROS production via lysosomes or by causing mitochondrial membrane permeabilization and producing mitochondrial ROS.

**Figure 3:**
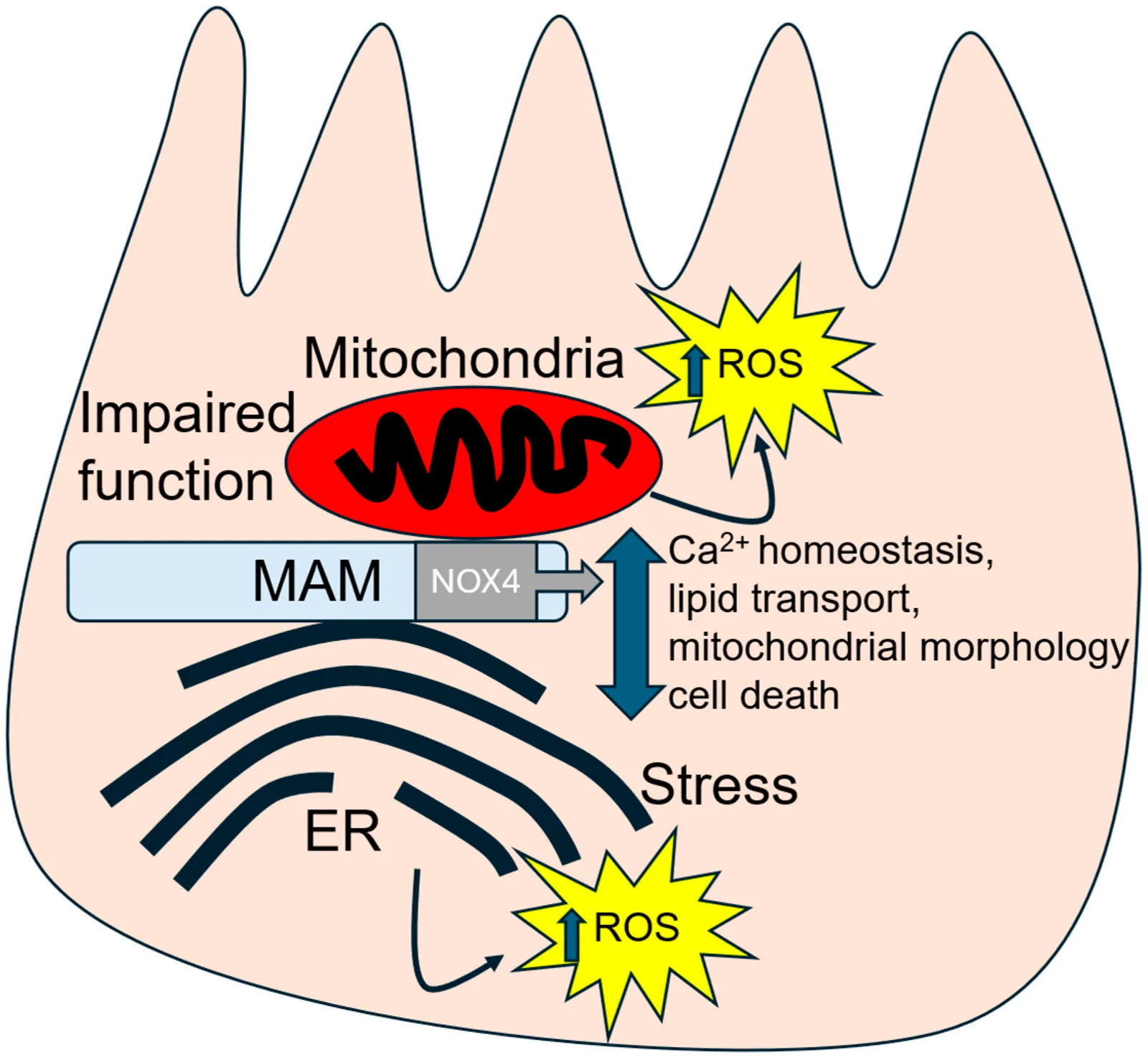
Mitochondria and ER are physically connected through mitochondria-associated membranes (MAMs). MAMs are a microdomain composed of specific proteins that play an important role in calcium homeostasis, mitochondrial morphology, lipid transport, and cell death. NOX4 has been reported to localize to MAMs, where it may play an important role as a redox sensor and signal transducer between the two organelles. In addition, elevations in NOX4 at the MAMs may negatively affect calcium homeostasis mediated by the InsP3 receptor, lipid metabolism, mitochondrial dynamics, and cell death (e.g., apoptosis and ferroptosis).

**Table 1:** Evidence from various model systems for ROS-induced ROS release (RIRR), a process by which one cellular component such as the mitochondria generates ROS to trigger additional release of ROS from another cellular compartment or organelle. RIRR can mediate intercellular communication and intracellular signaling. The accumulation of ROS eventually leads to cell death.

Model System	Important Findings	Reference
Cell-culture models	RIRR Initiation requires simultaneous induction of mitochondrial permeability transition (MPT) in cardiac myocytes. The corporative MPT/RIRR event occurs synchronously and reversibly among long chains of adjacent mitochondria.	[113]
	Nox2 senses Nox4-derived H_2_O_2_ to promote mtROS production as a novel mechanism RIRR in endothelial cells.	[114]
	Provided evidence that RIRR contributes to apoptosis in human malignant melanoma A375-S2 cells via extrinsic and intrinsic pathways.	[115]
	A reaction-diffusion model with RIRR showed the observed ROS transmission velocity in unicellular microorganism communities of cultured *C. reinhardtii*.	[116]
Animal/Human models	Shear stress within the vascular wall activates endothelial NADPH-oxidase in coronary artery disease patients, leading to RIRR.	[117]
Other models	Used a mathematical model to show that the macroscopic properties of the mitochondrial network can be reproduced in a reaction-diffusion model of RIRR.	[118]
	Engineered macrophage cell membrane-coated nanoparticles loaded with NLRP3 inhibitors were shown to inhibit cell pyroptosis and mitigate CaOx-induced renal injury.	[119]

## Data Availability

No new data were created or analyzed in this study. Data sharing is not applicable to this article.

## Abbreviations

The following abbreviations are used in this manuscript:

4HNE	4-hydroxy-2-nonenal
4-PBA	4-phenylbutyric acid
8-IP	8-isoprostane
ACE	angiotensin-1-converting enzyme
AKT	protein kinase B
ANG1	angiotensin I
AP-1	Activator Protein 1
ARB	AT1 receptor blockers
CaOx	calcium oxalate
CaP	calcium phosphate
COD	CaOx dihydrates
COM	CaOx monohydrates
CKD	chronic kidney disease
CSA	cyclosporine A
eNOS	endothelial nitric oxide synthase
ER	endoplasmic reticulum
ETC	electron transport chain
GAL	beta-galactosidase
G6PDH	glucose-6-phosphate dehydrogenase
GPx	peroxidase
GSH	glutathione
GST	glutathione S-transferase
H_2_O_2_	hydrogen peroxide
HIF1α	hypoxia-inducible factor 1α
HOCL	hypochlorous acid
HS	heparin sulfate
LMP	lysosome membrane permeabilization
MAMs	mitochondria-associated membranes
MAPK	mitogen-activated protein kinase
MDA	malondialdehyde
MGP	Matrix Gla Protein
MR	mineralocorticoid receptor
mPTP	mitochondrial permeability transition pore
NAG	N-acetyl-beta-glucosaminidase
NAC	N-acetyl cysteine
NADPH	nicotinamide adenine dinucleotide phosphate
NFκB	The nuclear factor kappa B
NLRP3	NOD-like receptor family pyrin domain-containing 3
NRF2	nuclear factor erythroid 2-related factor 2
OPN	osteopontin
OS	oxidative stress
OX	oxalate
PLA2	phospholipase A2
PI3K	phosphoinositide 3-kinase
PTF-1	prothrombin fragment-1
PKC	protein kinase c
RAS	renin-angiotensin system
RAAS	renin-angiotensin aldosterone system
RIRR	ROS-induced ROS release
TGF Beta1	transforming growth factor-beta1
THP	Tamm–Horsfall Protein
TXNIP	Thioredoxin-interacting protein
UPR	unfolded protein response
VIM	vimentin
